# The Controversial Role of Microglia in Malignant Gliomas

**DOI:** 10.1155/2013/285246

**Published:** 2013-07-24

**Authors:** Jun Wei, Konrad Gabrusiewicz, Amy Heimberger

**Affiliations:** Department of Neurosurgery, The University of Texas MD Anderson Cancer Center, Houston, TX 77030-1402, USA

## Abstract

Malignant gliomas contain stroma and a variety of immune cells including abundant activated microglia/macrophages. Mounting evidence indicates that the glioma microenvironment converts the glioma-associated microglia/macrophages (GAMs) into glioma-supportive, immunosuppressive cells; however, GAMs can retain intrinsic anti-tumor properties. Here, we review and discuss this duality and the potential therapeutic strategies that may inhibit their glioma-supportive and propagating functions.

## 1. Introduction

Microglia can constitute up to 10% of cells in the central nervous system (CNS) and are distinctive from other CNS cells such as astrocytes and oligodendrocytes [1]. Distinguishing features of microglia are their “ramified” branches that emerge from the cell body that communicate with surrounding neurons and other glial cells. Microglia rapidly respond to infectious and traumatic stimuli by adopting an “amoeboid” activated phenotype and can produce a variety of pro-inflammatory mediators such as cytokines, chemokines, reactive oxygen species (ROS), and nitric oxide (NO), which contribute to the clearance of pathogenic infections. However, prolonged or excessive activation may result in pathological forms of inflammation that contribute to the progression of neurodegenerative and neoplastic diseases [2].

**Figure 1:**
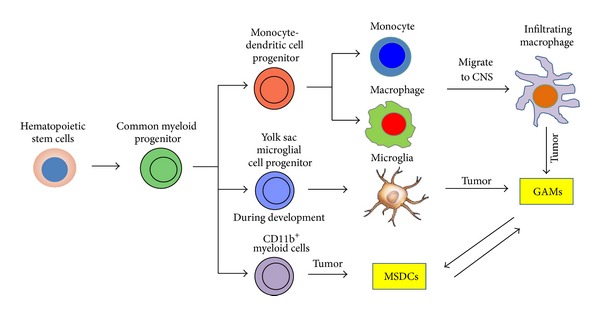
Cell lineage derivation of the CNS microglia/macrophage is depicted, with arrows indicating lineage relatedness. Myeloid-derived suppressor cells (MDSCs) are a lineage term describing glioma-associated microglia/macrophages (GAMs).

Gene transcript clustering analysis reveals that microglia have close lineage relationship with bone-marrow-derived macrophages, indicating that these cells arise from the bone marrow and circulating monocytes/macrophages [3]. However, recent findings suggest that microglia originate from yolk sac macrophages that migrate into the CNS during early embryogenesis [4] (Figure 1). Further confounding the issue regarding the origin of CNS microglia are studies demonstrating the generation of microglia-like cells from a murine embryonal carcinoma cell line (P19) during neural differentiation [5]. Regardless of the origin, the microglia/macrophage population are usually the dominant glioma-infiltrating immune cells (5–30%) [6].

Studies during the 90s demonstrated a positive correlation between the number of microglia/macrophage and glioma malignancy [7, 8]. Furthermore, microscopic analysis of microglia morphology in high-grade glioma revealed an activated state, described by amoeboid or spherical shape [9, 10]. Morioka et al. have found that reactive microglia form a dense band that surround the tumor mass and can extend along the corpus callosum into the contralateral cerebral hemisphere [11]. These data would indicate that microglia react to brain tumors; however, it remains to be determined whether this response represents an active anti-tumor defense mechanism or a tumor-supportive one. Likewise, microglia are commonly found “trapped” in gliomas and it is unclear if they have coproliferated with the tumor cells. Hepatocyte growth factor (HGF)/scatter factor (SF), which plays a role in glioma motility and mitogenesis, may be one chemokine responsible for the microglia infiltration in malignant gliomas [12]. Additionally or alternatively, monocyte chemotactic protein-3 (MCP-3) was found to correlate with GAM infiltration [13] and to act as a chemokine. Furthermore, it has been reported that tumor necrosis factor (TNF) dependent action enhances macrophage/microglia recruitment in glioma [14]. Other chemoattractants that have been shown to stimulate microglia/macrophage migration into the tumor include colony stimulating factor-1 (CSF-1) [15, 16], macrophage colony-stimulating factor (M-CSF) [17], and glial-derived neurotrophic factor (GDNF) [18]. However, recent studies have emphasized a predominant role of granulocyte-macrophage colony stimulating factor (GM-SCF) in microglia/macrophage attraction [19] which confirmed previous reports [20, 21]. Nonetheless, the increased number of microglia/macrophages found in high-grade gliomas has suggested that they may have a pro-neoplastic role [8].

No consensus definitive marker that distinguishs between microglia and macrophages has thus far been defined. As such, many investigators use the more general term “microglia/macrophages” instead of microglia alone. CD163, CD200, CD204, CD68, F4/80, and the lectin binding protein Iba-1 can be used as general markers of microglia/macrophages [22, 23]. P2X4R expression can define a distinct subset of GAMs [24]. Furthermore, allograft inflammatory factor-1 (AIF-1) and heme oxygenase-1 (HO-1) can also be used to define a distinct subset of GAMs in rat and human gliomas [25, 26]. However, the most commonly used criterion to distinguish CNS microglia from macrophages is the differential CD45 expression (CD45^low^ for microglia and CD45^high^ for macrophages) on CD11b^+^ CD11c^+^ cells [27–29]. The robustness of this is questionable.

Another confounding issue in defining and clarifying the biological role of GAMs is their distinction from myeloid-derived suppressor cells (MDSCs). The MDSCs are induced in response to various tumor-derived cytokines and have been shown to inhibit tumor-specific immune responses [30]. However, this is not an absolute since MDSCs isolated from mouse brain tumors, although expressing markers consistent with an immune suppressive phenotype (CCL17, CD206, and CD36), still express proinflammatory IL-1*β*, TNF-*α*, and CXCL10 [31]. Currently, there are two presumed MDSC populations: monocytic and granulocytic. Thus far little is known about the biology and phenotypic characteristics of MDSCs within gliomas but presumptively GAMs would be closer in the continuum to the monocytic MDSC subset based on CD11b^+^ expression and function. Specifically, MSDCs only weakly present glioma antigens to cytotoxic T cells and express FasL, which contributes to the local immunosuppressive milieu of malignant gliomas [32], similar immunological functions attributed to GAMs [33]. Furthermore, the MDSCs have been shown to inhibit T cell activity by NO production [34]; however, this immune suppression was postulated to be distinct from CNS microglia.

## 2. The M1/M2 Continuum

**Figure 2:**
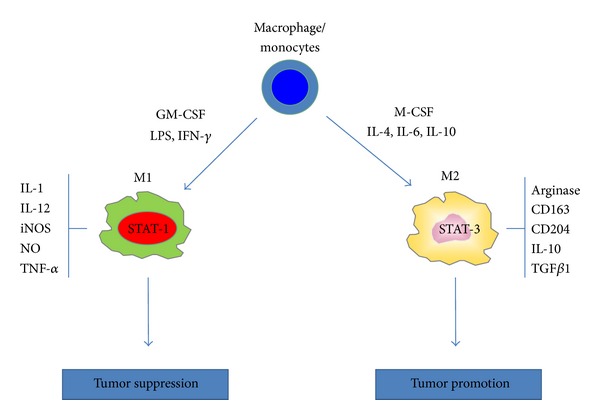
CNS macrophage/monocytes differentiate into polarized macrophage subsets when exposed to different cytokine milieu. In the presence of granulocyte-macrophage colony stimulating factor (GM-CSF), interferon-(IFN) *γ*, lipopolysaccharide (LPS) and other microbial products, monocytes differentiate into M1 macrophages. In the presence of macrophage colony stimulating factor (M-CSF), interleukin-(IL) 4, IL-6, IL-10 and immune suppressive molecules (corticosteroids, vitamin D3, prostaglandins), monocytes differentiate into M2 macrophages. M1 and M2 subsets differ in terms of phenotype and functions. M1 cells have high anti-microbial activity, immune stimulatory functions and tumor cytotoxicity and express the signal transducer and activator of transcription 1 (STAT-1). M2 cells have high scavenging ability, promote tissue repair and angiogenesis, favor tumor progression and express STAT-3.

**Figure 3:**
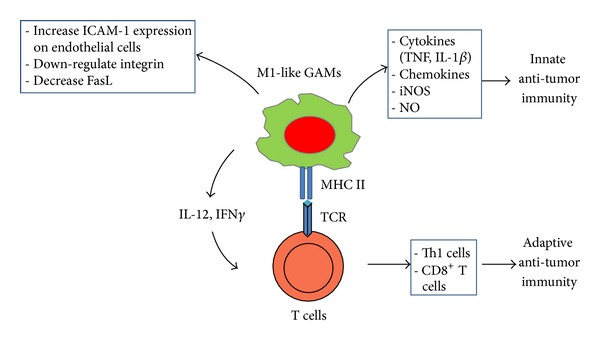
Glioma-associated microglia/macrophages (GAMs) have anti-tumoral potential. In certain circumstances, GAMs can be activated and polarized to a M1-like phenotype that can contribute to both innate and adaptive anti-tumor immunity.

**Figure 4:**
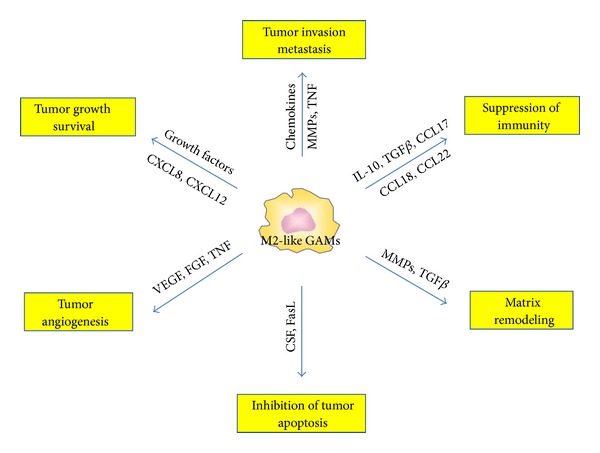
Glioma-associated microglia/macrophages (GAMs) are tumor supportive. Chemokines have a prominent role as they induce neoangiogenesis, activate matrix-metalloproteases (MMPs) and stroma remodeling, and direct tumor growth. Selected chemokines and immunosuppressive cytokines inhibit the anti-tumor immune response.

The M1/M2 continuum has been applied to CNS infiltrating macrophage/monocytes in the context of inflammation or tumor. Classically activated macrophages assume a M1 phenotype characterized by the expression of the signal transducer and activator of transcription 1 (STAT-1) and the production of iNOS (Figure 2). The M1 cell is capable of stimulating anti-tumor immune responses by presenting antigen to adaptive immune cells, producing pro-inflammatory cytokines and phagocytosing tumor cells [35] (Figure 3). Whereas the alternatively activated pathway, M2, is characterized by the expression of surface CD163 and CD204, expression of intracellular STAT-3 and the production of arginase [36, 37] (Figure 2). M2 polarization prevents the production of cytokines required to support tumor-specific CD8^+^ T cells, and CD4^+^ T helper 1 (Th1) and promotes the function of CD4^+^ regulatory T cells, and are therefore tumor supportive [38, 39] (Figure 4). Multiple lines of evidence suggest that the GAMs are likely skewed to the alternatively activated M2 macrophage phenotype. However, to date, it should be noted that a comprehensive characterization of the M1 and M2 composition of gliomas has not been conducted.

Glioma cells secrete a wide variety of factors that suppress immune cells, such as IL-10, IL-4, IL-6, M-CSF, macrophage inhibitory factor (MIF), TGF*β*, and prostaglandin E2 (PGE2) [17, 40–43]. These cytokines are known to promote a M2 phenotype and/or to suppress the M1 phenotype. For example, TGF*β* inhibits microglia cell proliferation and the production of proinflammatory cytokines *in vitro* [44]. IL-4, IL-6 and IL-10 have been shown to polarize microglia to an M2-like phenotype [45]. Other immunosuppressive mechanisms such as the downregulation ICAM-1 and expression of immune inhibitory molecules such as B7-H1 can also dismantle the microglia-T cell combined immune recognition and clearance of gliomas [46]. Furthermore, gliomas induce upregulation and expression of HLA-G and HLA-E by GAMs in a majority of glioblastomas, thus hindering anti-glioma activity [47]. The anti-glioma functional impairment of GAMs likely occurs relatively late in the course of glioblastoma tumor growth, potentially providing a window of opportunity for therapeutic strategies directed towards preventing their functional impairment [48]. Of note, the M1/M2 distinction is a continuum and simplification of the complex T cell and GAM interactions and the subsequent outcome.

Similar impairments of microglia/macrophage anti-tumor activity have been reported for brain metastasis. In the region where the tumor mass is situated, only a few microglia express inducible nitric oxide synthase (iNOS) and tumor necrosis factor-alpha (TNF-*α*). On one hand, the microglia accumulate as a result of migration and proliferation, have an ameboid appearance, and appear to actively respond to the metastatic lung cancer cells in the brain by tightly encapsulating the tumor mass. On the other hand, the lack of positivity of either iNOS or TNF-*α* in double-labeling experiments indicates that the microglia immunological functions including cytotoxicity, phagocytosis, and antigen presentation are impaired [49]. Microglia have also been shown to promote invasion and colonization of the brain by breast cancer by serving both as active transporters and guiding rails. This is antagonized by inactivation of microglia as well as by Wnt signaling inhibition. Therefore, microglia were shown to be critical for the successful colonization of the brain by epithelial cancer cells, suggesting that inhibition of microglia could also be a promising anti-metastatic strategy [50].

Despite the immunosuppressive environment of human gliomas, GAMs are capable of some innate immune responses such as phagocytosis, cytotoxicity and TLR expression but are not competent in secreting key pro-inflammatory cytokines [51]. GAMs responsiveness to activators is impaired when compared to microglia/macrophages isolated from normal brain; specifically, the former has an impaired capacity to be stimulated by TLR agonist to secrete cytokines, upregulate costimulatory molecules, and activate anti-tumor effector T cells [33]. Understanding the mechanism of these differences may be critical in the development of novel immunotherapies for malignant gliomas [52].

The GAMs do not always display MZ functions. Insoluble matrix components derived from malignant glioma cells have been shown to enhance microglia activation [30]. These activated microglia can then induce glioma cell death by blockade of basal autophagic flux inducing secondary apoptosis/necrosis [53]. Signaling through the JAK-2-STAT-5 pathway was shown to be essential for IL-3-induced activation of microglia [54]. Furthermore, microglia/macrophages depletion increased glioma tumor volume by 33%. This was not believed to be secondary to the loss of microglia tumoricidal activity because phagocytosis or apoptosis of glioma cells was rarely observed. The loss of anti-tumor effect was also not due to alterations in tumor vasculature. Rather, the investigators found that depletion of a subset of CD86^+^ microglia with M1-like characteristics could present alloantigen and secrete stimulatory cytokines that promoted the expansion of the CD8^+^ T cells and was likely the etiology for enhanced glioma growth [55].

Alternatively, M2-like GAMs also promote glioma growth/survival via enhancing angiogenesis and inhibiting tumor apoptosis. GAMs have been shown to release growth factors such as VEGF, PDGF, and members of the FGF family, and a correlation has been shown between GAM numbers and tumor vascularity in gliomas [56]. Furthermore, these growth factors sustain malignant cell survival and subsequent tumor growth. Lastly, GAMs are a major source of FasL expression, which likely contributes to the suppression of malignant glioma apoptosis [32].

Glioma regression has also been correlated with greater numbers of T cells and microglia, suggesting that the combined mobilization of peripheral and CNS endogenous immune cells is required for eradicating large intracranial tumors [57]. Some soluble factors from reactive microglia are capable of enhancing the expression of ICAM-1 on the brain endothelial cells (ECs). As a consequence, large numbers of tumor-primed T lymphocytes can adhere to EC and migrate across the EC monolayer [58]. Finally, antibody-dependent cell mediated cytotoxicity (ADCC) has also been shown to be involved in microglia anti-tumor activities. Microglia derived from brain cortices of newborn mice were shown to lyse human tumor cell lines expressing different levels of epidermal growth factor receptor (EGFR) in the presence of a monoclonal antibody specific to EGFR [59]. Reconciliation of the cumulative data would indicate that GAMs can be either M1 or M2 or some were in between, but the functional outcome will depend on the relative composition of M1 and M2 cells within the glioma.

## 3. Interplay between GAMs and Gliomas

It is becoming apparent that crosstalk exists between the GAMs and the brain tumor cells. Gliomas promote the recruitment, proliferation, and M2 polarization of microglia/macrophages; reciprocally, GAMs facilitate the survival, growth, and especially the spread of glioma cells (Figure 4). When activated in the presence of glioma, microglia invade the tumor. The microglia then secrete a variety of factors that degrade the CNS matrix. The glioma cells simultaneously invade and expand into the dissociated tissue by utilizing the same corridor paved by laminin on astrocytic projection for microglia invasion into the tumors [60–62]. Glioma secreted factors, by engaging the toll-like receptors and the p38 MAPK pathway, trigger the expression and activity of membrane type 1 metalloprotease (MT1-MMP) on GAMs. The GAM MT1-MMP expression then in turn activates glioma-derived pro-MMP-2 that subsequently promotes glioma invasion. A deficiency of MyD88, an upstream mediator of MT1-MMP, or microglia depletion, largely attenuates glioma expansion *in vivo* [62]. Furthermore, in brain slices inoculated with glioma cells, increased activity of metalloprotease-2 was directly correlated with the abundance of microglia [63]. Another GAM-associated mechanism, the CX3CR1/CX3CL1 interaction, can also induce MMPs production resulting in glioma invasion [64]. A recently described factor, STI1 (cochaperone stress inducible factor 1) secreted by microglia was shown to favor tumor growth and invasion through the participation of MMP-9 [65]. Thus, glioma cells stimulate microglia to increase the breakdown of extracellular matrix, thereby, promoting glioma invasion.

Substantially more controversial is whether microglia initiate or participate during early gliomagenesis. Neurodegeneration, neurotoxicity, and neuroinflammation are associated with chronic microglia activation that has been postulated to contribute to gliomagenesis [66–68]. Cyclooxygenase-1 (COX-1) in microglia/macrophages might represent a key regulatory mechanism in the inflammatory processes associated with neoplasia [69]. COX-1 and COX-2 differential accumulation is observed in microglia/macrophages and astrocytes during oligodendroglioma progression *in vivo* [70]. Furthermore, induction of COX-2 in microglia contributes to the deleterious effects of prostanoids in cerebral edema formation during the progression of oligodendrogliomas [71]. Increased c-Jun-NH2-kinase signaling in neurofibromatosis-1 heterozygous (Nf1^+/−^) microglia was shown to promote optic glioma proliferation [72] and glioma growth [73]. Interestingly, IL-4 or IFN-*γ*-mediated microglia activation differentially induces oligodendrogenesis and neurogenesis, respectively, from adult stem/progenitor cells. It thus appears that how microglia are activated determines their ability to either support or impair cell renewal and differentiation from adult stem cells [74].

Glioblastomas are believed to arise from glioma stem cells (GSCs). GSCs are phenotypically similar to normal stem cells, can express CD133, and possess self-renewal potential [75]. GSCs recapitulate the original polyclonal tumors when xenografted into nude mice and mediate chemo- and radiation resistance, thereby, leading to tumor progression and recurrence. A positive correlation is found between the degree of infiltration of GAMs and the density of GSCs. The capacity of GSCs to recruit GAMs was found to be stronger than glioma cell lines indicating that the GSCs play a predominant role in microglia/macrophages tropism to glioma [76]. In addition, recent mechanistic studies by another group showed that TGF*β*1 released by GAMs promoted the expression of MMP-9 by GSCs, and TGFR2 knockdown reduced the invasiveness of these cells *in vivo *[77].

We have previously shown that GSCs produce a variety of cytokines known to recruit and polarize the microglia/macrophages to become immunosuppressive. The GSC-conditioned medium polarized the microglia/macrophage to an M2 phenotype, inhibited microglia/macrophage phagocytosis, induced the secretion of the immunosuppressive cytokines IL-10 and TGF*β*1 and enhanced the capacity of these cells to inhibit T-cell proliferation [44]. The inhibition of antigen-presenting capabilities of GAMs by glioma tumor cells has also been demonstrated [25]. Previously, glioma-derived M-CSF was shown to induces markers reflective of the M2 phenotype CD163 and CD204 on monocytes, and in turn these differentiated microglia/macrophage facilitated tumor growth. Both the extent of M-CSF production and CD163^+^ and CD204^+^ expression on microglia were correlated with glioma grade [17]. A direct correlation has been shown between the immunogenicity of glioma tumor cells and the GAM content and their antigen-presenting function [78, 79]. In addition, the metabolic status of the microglia is altered by the glioma environment [80]. Abundant amounts of ATP released by the glioma have been suggested to trigger P2X_7_R signaling, resulting in increase of MIP-1*α* (macrophage inflammatory protein-1*α*) and MCP-1 (monocyte chemoattractant protein-1) in GAMs [81]. Cumulatively these data indicate that there is a strong interaction that occurs between the GAM and glioma that ultimately influences the biological behavior of each one.

## 4. Therapeutic Manipulation of the GAM

A variety of therapeutic anti-glioma strategies have suggested that modulation of the GAM population may contribute to therapeutic efficacy. Inhibition of experimental rat glioma growth by decorin (TGF*β* antagonism) gene transfer was associated with decreased microglia infiltration suggesting that the GAMs were participate in the regression of decorin-expressing rat C6 gliomas [82]. Recent studies have demonstrated that ablation of CD11b^+^ cells in ganciclovir-treated CD11b-HSVTK mice [83] or *in vivo* targeting folate receptor *β* (FR*β*)-expressing tumor-associated macrophages, decreases tumor size and improves animal survival [84]. The presence of significant anti-tumor immunity following herpes simplex virus 1 thymidine kinase (HSV-TK) and ganciclovir (GCV) treatments suggests that the immune system plays a critical role in the sustained tumor regressions associated with these treatments. Histologic examination of the brains of the successfully treated animals demonstrated residual tumor cells and inflammatory cells consisting predominantly of macrophages/microglia and T cells [85]. Another report demonstrates that a reduction of peripheral CD163^+^ macrophages *in vivo* and the depletion of CD68^+^ macrophage/microglia within brain slice *ex vivo* increase the intratumoral oncolytic viral titer to 5-fold and 10-fold, respectively, [86].

Therapeutic targeting of the GAM could be directed toward their activation (Table 1). Clearly, the goal of therapeutically targeting GAMs would be to selectively inhibit M2 while enhancing M1 functions (Table 2). The latter approach would include strategies that (1) inhibit the molecular mechanisms used by M2 cells to block lymphocyte reactivity and proliferation; (2) induce M2 apoptosis and/or trafficking to the tumor; or (3) force GAMs to the M1 phenotype. Inhibition of the M2-like activation of tumor-infiltrating macrophages was shown to significantly reduce glioma growth [37]. As such, STAT-3 is an attractive candidate since this pathway mediates glioblastoma-mediated M2 skewing and immune suppression. STAT-3 blockage by WP1066 stimulates the immune activation of GAMs, as evidenced by their increased expression of costimulatory molecules CD80 and CD86 [117]. Furthermore, *in vivo* STAT-3 inhibition in murine GAMs was shown to reduce expression of immunosuppressive cytokines, such as IL-10 and IL-6, while stimulating production of pro-inflammatory TNF-*α* [118]. Another therapeutic option is glycoprotein T11TS, which has been found to upregulate MHC class II, CD2 and CD4 expression in microglia *in vivo* in a rat glioma model [40]. Because macrophages/microglia express the nicotinic acetylcholine receptor (AchR) on their surface, a short AchR-binding peptide derived from the rabies virus glycoprotein (RVG) could also be used for targeted delivery of siRNA to macrophages for anti-tumor treatment. This peptide was fused to nona-D-arginine residues (RVG-9dR) to enable siRNA binding [124]. Recently, GAMs were shown to enhance GSCs' invasion via the TGF*β*1 signaling pathway [77]. shRNA against TGF*β*1 receptor (TGF*β*R) on tumor-associated macrophages strongly inhibited glioma invasiveness, indicating that TGF*β*R is a potential therapeutic target [112]. Several small molecules exist with TGF*β*R inhibitor activity that could be utilized as GAM modulatory agents. Studies of Carpentier et al. [87] revealed that intratumoral injections of oligodeoxynucleotides containing CpG motifs (CpG-ODN) trigger both innate and specific immunities, driving the immune response towards the Th1 phenotype. On the other hand, glioma-bearing animals treated with minocycline showed increased survival [125] and reduced glioma invasion by attenuated microglia MT1-MMP expression [106].

It should be noted that glioblastoma-mediated immune suppression is notoriously heterogeneous and plastic. Thus, targeting the M2 population may only result in a therapeutic response in a subset of patients in which this mechanism is operational. Furthermore, the therapeutic response of M2 targeting may be limited when other immune suppressive mechanisms are appropriated or upregulated by the tumor. However, the suppression/inhibition of glioma-derived factors or glioma-mediated immune suppression has been shown to synergize with the efficacy of microglia therapeutic strategies [118, 126–129], suggesting that there is a therapeutic opportunity.

 GAMs or their precursors could be used to facilitate CNS tumor imaging [130]. Accurate delineation of tumor margins is vital to the successful surgical resection of brain tumors and the extent of resection impacts survival. The nanoparticle CLIO-Cy5.5 is taken by microglia and is detectable by both magnetic resonance imaging and fluorescence. It could be used to assist intraoperatively in visualizing tumor boundaries because CD11b^+^ microglia are found at the tumor margin [131]. Other labeling system include cyclodextrin-based nanoparticles (CDP-NPs) [132] or multiwalled carbon Nanotubes (MWCNTs) [133]. Recently, quantum dots have been shown to be phagocytized by microglia and macrophages that infiltrate experimental gliomas that resulted in improved identification and visualization of tumors, potentially augmenting brain tumor biopsy and resection [134]. Ultimately, these imaging approaches could be exploited as biomarkers to monitor clinical trials targeting the GAM population [135].

## 5. Summary

The data indicates that GAMs may not solely inhibit or enhance glioma growth. Rather, the dominant propensity depends on the interacting microenvironment. It is possible that the tumor-supportive role of the microglia is unintentional—a tumor subversion of a physiological response normally used to deescalate immunological reactivity. Typically the CNS microglia are providing a surveillance function of CNS tissue, guiding the lymphocytes there and exerting their own effector functions and scavenging to wipe out glioma cells. In the early stages of gliomagenesis, innate responses mediated by microglia may be beneficial and involve the activation of effective surveillance by adaptive immunity resulting in the elimination of these cells [136]. However, in the context of progressive malignancy, when tumor cells have escaped immune editing, the smoldering inflammation orchestrated by GAMs, may promote tumor progression. Therapeutic strategies targeting macrophages should take into account the dual role of these cells.

## Figures and Tables

**Table 1:** Microglia activating agents.

Molecule/agent	Classification	Mechanism/action	Reference
CpG-ODN	TLR9 ligand	Increases microglia tumor infiltration and enhances the antigen-presenting capacity	[87–89]
poly (I:C)	TLR3 ligand	Unknown soluble factors	[90]
IL-12	Th1 cytokine	Increases tumor infiltration and enhances TRAIL and phagocytosis	[91, 92]
TNF	Th1 cytokine	Enhances glioma cytotoxicity	[93]
IFN-*γ*	Th1 cytokine	Upregulates class II MHC antigen expression	[94]
Cytotoxic T cells	Immune cells	Induce microglia activation and recruitment	[95, 96]
C1q, complement receptor 3 (CR3)	Complement	Mediates elimination of tagged synapses and activates microglia	[97, 98]
T11TS/SLFA-3	Glycopeptide	Induces MHC class II expression and facilitates SLFA3/T11TS-CD2 immune activation	[99, 100]
Ceramide	Sphingolipid	Enhances microglia production/secretion of brain-derived neurotrophic factor (BDNF)	[101]
Ganglioside	Glycosphingolipid	Activates microglia via protein kinase C and NADPH oxidase, which regulate activation of NF-*κ*B	[102]
Adenosine	Nucleoside	Acts via A1 adenosine receptors in microglia	[60]
Triggering receptor expressed on myeloid cells-2 (TREM2)	Innate immune receptor	Increases phagocytosis	[103]
Prothrombin	Blood-clotting protein	Activates microglia via kringle-2 domain	[104]
Propentofylline (PPF)	Methylxanthine	Inhibits microglia migration toward tumor cells and decreases MMP-9 expression	[105]
Minocycline	Antibiotic	Reduces glioma expansion and invasion by attenuating microglia MT1-MMP expression	[106]
Cyclosporin (CsA)	Immunosuppressant	Inhibits immunosuppressive microglia via MAPK signaling	[107]
Mifamurtide	Muramyl dipeptide	Enhances macrophage cytotoxicity and has been used for osteosarcoma treatment	[108]
Butyrate	Fatty acid	Anti-inflammatory in primary, brain-derived microglia cells, but is proinflammatory in transformed, proliferating N9 microglia	[109]
I-125	Radioactive isotope	Stimulates microglia/macrophage to remove necrotic debris	[110]

**Table 2:** Molecules/targets in GAMs for therapeutic modulation.

Molecule/agent	Classification	Mechanism	Reference
CSF-1R	Cytokine receptor	Inhibits glioblastoma invasion by targeting glioblastoma-associated microglia via inhibition of the CSF-1R	[16, 111]
TGF*β*1	Cytokine	GAMs enhance the invasion of GSCs via TGF*β*1 signaling pathway, which increases the production of MMP-9 by GSCs	[77, 112]
IL-4	Cytokine	Inhibits inflammatory mRNA expression in mixed rat glial and in isolated microglia cultures	[113]
IL-16	Cytokine	Expression correlates with WHO grades of human astrocytic brain tumors	[114]
MCP-1	Cytokine	A positive amplification circuit for macrophage recruitment in gliomas	[115]
Vasoactive intestinal peptide (VIP) and pituitary adenylate cyclase-activating polypeptide (PACAP)	Neuropeptides	Inhibit the production of inflammatory mediators by activated microglia, thus, defined as “microglia-deactivating factors”	[116]
STAT-3	Transcription factor	STAT3 inhibition activates tumor macrophages and abrogates glioma growth	[44, 117–119]
Cyclophosphamide (CPA)	Alkylating agent	Pretreatment with CPA inhibits an increase of CD68^+^ and CD163^+^ cells and therefore enhances HSV replication and oncolysis	[120]
Dexamethasone	Steroid	Inhibits the filtration of microglia into brain tumors	[121]
ATP	Nucleotide	Promotes an anti-inflammatory state in both hematogenous and resident myeloid cells of the CNS	[122]
Radiochemotherapy	Therapy	Depletes CD68^+^ microglia	[123]

## Abbreviations

AchR: Acetylcholine receptor
ADCC: Antibody dependent cell mediated cytotoxicity
AIF-1: Allograft inflammatory factor-1
APC: Antigen-presenting cell
CpG-ODN: CpG-oligodeoxynucleotide
CNS: Central nervous system
DcR3: Decoy receptor 3
EC: Endothelial cells
EGF: Epidermal growth factor
GAMs: Glioma-associated microglia/macrophages
GSCs: Glioma cancer stem cells
HGF: Hepatocyte growth factor
HO-1: Heme oxygenase-1
HSV-TK: Herpes simplex virus 1 thymidine kinase
IL: Interleukin
iNOS: Inducible nitric oxide synthase
MDSCs: Myeloid-derived suppressor cells
MHC: Major histocompatibility complex
MIF: Macrophage inhibitory factor
MMP: Matrix metalloproteinase
NO: Nitric oxide
ROS: Reactive oxygen species
PACAP: Pituitary adenylate cyclase-activating polypeptide
PGE2: Prostaglandin E2
SF: Scatter factor
STAT: Signal transducer and activator of transcription
TGF: Transforming growth factor
TLR: Toll-like receptor
TMZ: Temozolomide
TNFR: Tumor necrosis factor-related
Tregs: Regulatory T cells
VEGF: Vascular endothelial growth factor
VIP: Vasoactive intestinal peptide.
